# Point‐of‐Care Ultrasound in Undergraduate Medical Education: A National Survey

**DOI:** 10.1002/jum.70021

**Published:** 2025-08-04

**Authors:** Frances M. Russell, Reena Park, Molly Campbell, Laura Lemen

**Affiliations:** ^1^ Department of Emergency Medicine Indiana University School of Medicine Indianapolis Indiana USA; ^2^ Indiana University School of Medicine Indianapolis Indiana USA

**Keywords:** medical education, national survey, ultrasound

## Abstract

**Objective:**

The primary aim of this study was to assess the state of point‐of‐care ultrasound (POCUS) integration in undergraduate medical education (UME) at medical schools in the United States.

**Methods:**

This was a cross‐sectional survey study on POCUS implementation at 155 allopathic medical schools. The survey, which was distributed to Clinical Ultrasound Directors and medical school curricular deans, consisted of 18 questions and collected data on school characteristics and POCUS curricula details.

**Results:**

The survey response rate was 84% (130/155). Data showed that 66% (84/130) of schools had an approved POCUS curriculum, and 13 had a 4‐year longitudinal curriculum. Most schools required POCUS education in the preclinical years. Curricula taught a broad range of modalities for diagnostic POCUS, with focused assessment with sonography in trauma and cardiac being the most taught exams. Forty‐nine schools evaluated students' progress and understanding of POCUS.

**Conclusions:**

When compared to 5 years ago, more schools are implementing POCUS in UME, with 84 having an approved curriculum. Few schools offer a longitudinal curriculum, and only 49 are assessing students' POCUS knowledge.

**Abbreviations**
POCUS: point‐of‐care ultrasound
UME: undergraduate medical education

Point‐of‐care ultrasound (POCUS) has become increasingly important in doctor's offices, emergency departments, and hospital wards due to its non‐invasive nature and ease of use.^1^ As POCUS use continues to expand into more areas of medicine, it becomes increasingly vital that medical education does not fall behind. POCUS is user dependent and although is faster and less expensive than radiography, the accuracy of the examination relies on education and hands on training that the user received previously.^1^

When initially evaluated by a national survey in 2012, it was found that 51 allopathic medical schools in the United States offered optional or required POCUS training, and POCUS was most commonly taught in the third year of medical school education.^2^ A survey conducted just 2 years later found that only 48 schools had implemented a required POCUS curriculum for all students that was most commonly integrated into the preclinical curriculum.^3^ Both of these previous surveys found barriers for implementation of a POCUS curriculum, which included lack of funding for equipment and faculty, and lack of time in the current medical school curricula.^2^, ^3^ The most recent study evaluating POCUS teaching in undergraduate medical education (UME) was conducted by our study team in 2020, and found that 69 schools had an approved POCUS curriculum, with 10 requiring POCUS in all 4 years of training.^4^

As POCUS utilization continues to increase in medical specialties and in UME, we set out to determine the current state of POCUS training in allopathic medical schools in the United States. We hypothesized that more programs across the country have implemented POCUS training programs over the last 5 years.

## Methods

### Study Design

This was a cross‐sectional survey of Clinical Ultrasound Directors and medical school curricular deans from allopathic medical schools in the United States conducted from December 2024 to February 2025. The study was approved by the local Institutional Review Board and deemed exempt.

### Survey Design

The survey was initially developed in Qualtrics (Qualtrics LLC, Provo, UT, USA) and field tested in 2019.^4^ For this study, we simplified the survey and decreased from 6 to 4 sections, see online supplemental Appendix 1. We collected demographic information from the person filling out the survey and the schools, including the number of campuses. The survey gathered information on the existing POCUS curriculum at each school, including whether the curriculum was approved by the school's curriculum committee, whether the curriculum was required or optional, in what years the curriculum was being taught, what POCUS topics were being taught, what specialties were involved in the POCUS education, and if learners were assessed on their POCUS knowledge and skills.

### Participants

The survey was distributed by email to Clinical Ultrasound Directors and medical school curricular deans from 155 allopathic medical schools that had Liaison Committee on Medical Education accreditation. From a list of medical schools on the Liaison Committee on Medical Education website, we identified Directors of Clinical Ultrasound in UME who were the primary target audience for the survey as they would be integral to POCUS integration at their school and the most knowledgeable about the nuances of the curriculum. If we could not identify the Director of Clinical Ultrasound, then we contacted people in the following order: the Ultrasound Division Director in the Department of Emergency Medicine, Ultrasound Faculty, and Dean of Education and Curriculum. If we received multiple responses from a single institution, then we used the Clinical Ultrasound Director's response.

To identify the Clinical Ultrasound Director or the person most knowledgeable at each institution regarding the POCUS curriculum we started by using the American Institute of Ultrasound in Medicine website. For schools not listed in this directory we identified POCUS faculty through the Society for Clinical Ultrasound Fellowships website and Academic Emergency Ultrasound listserv. If we still could not identify a point person for the survey, we browsed the emergency medicine faculty directory on each institution's webpage to identify faculty who listed POCUS or ultrasound education as an interest or publication topic. Additionally, a web search for the institution name appearing with “point‐of‐care ultrasound” was conducted to identify a faculty contact.

In cases where we did not identify a POCUS contact, we emailed the Office of the Dean of Education or equivalent to obtain contact information for a POCUS director for UME where possible, or if not possible, we asked the Dean to complete the survey.

In December of 2024, we sent an invitation to participate in the survey via Qualtrics, which included a letter explaining the purpose of the survey. We also gave away one $50 gift card to increase participation. We sent weekly reminders from Qualtrics in addition to targeted emails to POCUS Directors or Deans. A representative from each institution was contacted a minimum of five times. If we did not receive a response from an institution either by survey or email communication, then we excluded those institutions from further analysis.

### Data Analysis

A descriptive data analysis was performed using frequency and percentage distributions. Data were analyzed with Microsoft Excel (Microsoft, Redmond, WA, USA).

## Results

**Figure 1:**
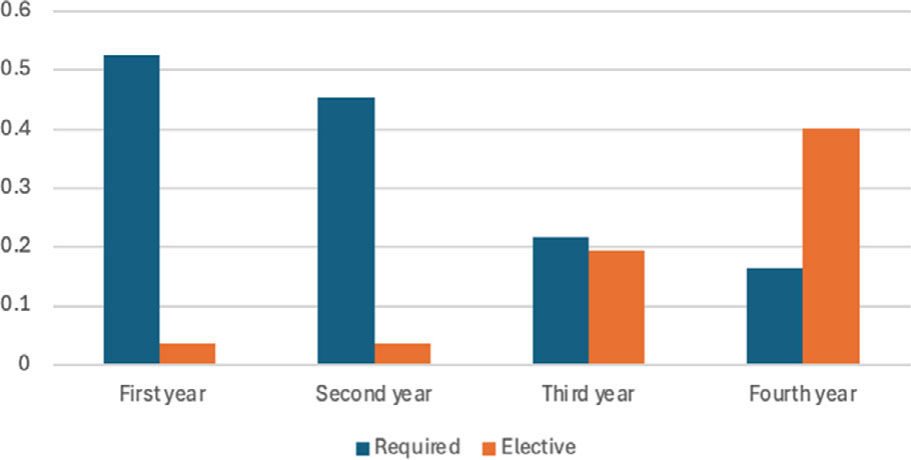
Required and elective point of care ultrasound teaching at allopathic medical schools in 2024 broken down by medical school year.

From the 155 medical schools surveyed, we received responses from 130 (84%). Of the respondents, 86 out of the 130 (66%) schools had an approved POCUS curriculum, while 44 schools did not. Forty‐three schools (33%) were multi‐campus, and 28 out of 43 (65%) schools had an approved curriculum. Eighty‐seven schools had only one campus and 58 (67%) had an approved POCUS curriculum. Of the 86 schools with an approved POCUS curriculum, 75 (87%) had required elements, while 11 had only elective or optional elements in the curriculum. The majority of medical schools with an approved POCUS curriculum required training in the first and second years of medical school, while elective or optional opportunities were offered more frequently during the third and fourth years of medical school, see Figure 1.

**Figure 2:**
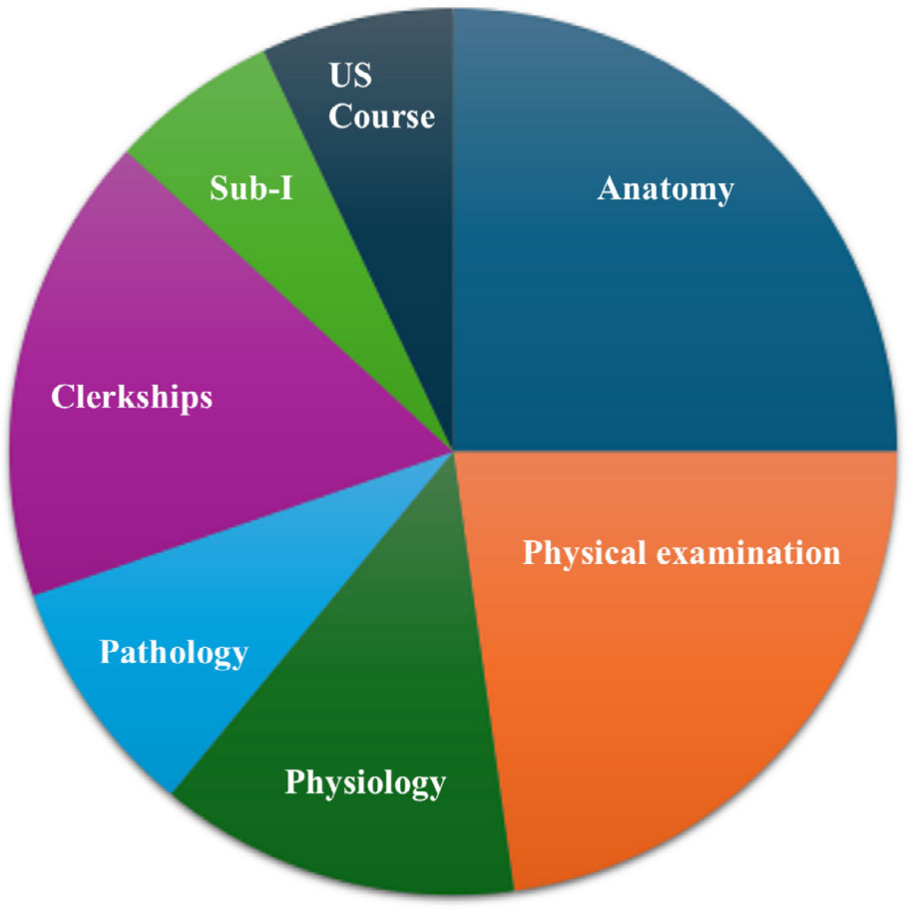
Allopathic medical school courses in 2024 that required ultrasound training.

Of the schools with an approved curriculum, 15% (13/86) had a longitudinal curriculum, where POCUS training was required in all 4 years. Required POCUS training was most commonly integrated into anatomy (41% of schools), physical examination (37%) and clerkships (28%); see Figure 2.

Medical schools were teaching a broad range of modalities for diagnostic POCUS, with focused assessment with sonography in trauma and cardiac being the most commonly taught POCUS exams. Only 50 (58%) schools were teaching procedural POCUS, with vascular access being the most taught; see Table 1.

**Table 1:** POCUS modalities being taught at allopathic medical schools in 2024

	*n* (%)
Diagnostic	
Aorta	53 (61.6%)
FAST	64 (74.4%)
Cardiac	65 (75.5%)
Gallbladder	58 (67.4%)
Kidney	58 (67.4%)
Lung	53 (61.6%)
MSK	47 (54.7%)
Obstetric	41 (47.7%)
Soft tissue	41 (47.7%)
Ocular	32 (37.2%)
RUSH	40 (46.5%)
Procedural	
Vascular access	50 (58.1%)
Paracentesis	15 (17.4%)
Thoracentesis	13 (15.1%)
Nerve blocks	12 (14.0%)

FAST, focused assessment with sonography in trauma; MSK, musculoskeletal; RUSH, rapid ultrasound in shock and hypotension; POCUS, point‐of‐care ultrasound.

The most common specialties teaching POCUS in the UME curriculum at each institution were emergency medicine (94%), internal medicine (48%), critical care (47%), family medicine (31%), and radiology (25%). Only six schools reported using ultrasound technologists. We were missing data from 22 schools, where this section was left blank.

Seventy‐four (59/80) percent of schools were using handheld POCUS machines to implement their curriculum, with seven exclusively using handheld machines and not a combination of handheld and cart‐based machines. We were missing data from 6 schools, where this section was left blank.

Sixty‐one (49/80) percent of schools were evaluating students' progress or understanding of POCUS, while 31 schools were not. Knowledge was being assessed through test questions (42/47, 89%), review of submitted POCUS images (22/47, 47%), and hands‐on assessments (30/47, 30%). Psychomotor skill was being assessed through simulated cases (9/47, 19%), review of submitted images (24/47, 51%), and objective clinical structured evaluations (29/47, 62%).

## Discussion

POCUS use continues to grow in medicine due to its portability and ability to make a rapid diagnosis or guide a clinical procedure.^5^ Training in UME and graduate medical education increasingly becomes more important to prepare medical students and residents for real‐world practice.^6^, ^7^ Inclusion of POCUS in UME has been found to improve student knowledge of anatomy and improve performance in clerkships.^6^, ^7^ In this study, we found that 66% of schools had an approved POCUS curriculum and 13% of those schools had a longitudinal 4‐year curriculum.

When comparing these results from prior data, we see an increase in the number of schools implementing an approved POCUS curriculum, from 51 in 2012^8^ to 69 in 2020^10^ to 86 in this current study. There are multiple possible reasons for this increase in implementation. For one, many specialties now have POCUS requirements in residency training.^8^, ^9^, ^10^ Starting this training earlier can help prepare medical students for residency and fellowship training. A previous survey showed that 97% of survey respondents, consisting of POCUS directors and Curricular Deans, reported that POCUS should be taught in UME. This was inclusive of those who already had a UME POCUS curriculum and those that did not.^4^ Also, multiple institutions expressed interest or were already planning to implement a POCUS curriculum in 2020.^4^ Additionally, multiple schools were using handheld POCUS machines to teach. Handheld machines can perform similarly to traditional cart‐based machines, are smaller and more mobile, fitting into a pocket, and are significantly cheaper, making them a lower cost alternative to cart‐based machines, which has been described as a barrier to POCUS implementation in UME.^2^, ^3^, ^4^ While this may or may not have impacted medical schools implementing POCUS in UME, the COVID‐19 pandemic highlighted the utility of POCUS for diagnosis and monitoring disease progression, particularly for lung ultrasound.^11^

Compared to 5 years ago, we saw an increase in the number of schools with a longitudinal 4‐year curriculum going from 10 to 13 schools. This is a smaller increase than expected but potentially a reflection of the amount of time and resources needed to provide education longitudinally. While we did not specifically address barriers in this study, to limit the number of questions in the survey and the time needed to take it, prior studies have found many limitations to implementing POCUS in UME, including lack of trained faculty, lack of time in the current curriculum, lack of protected time to design and implement a curriculum, and lack of equipment.^2^, ^3^, ^4^ The drop‐off in POCUS education from preclinical to clinical years is an important finding, as data has shown that POCUS skills significantly decline in as little as 8 weeks of non‐use.^12^, ^13^ Future studies should look to address specific barriers and facilitators for implementing POCUS in UME across all 4 years of training by conducting structured interviews with key stakeholders ranging from students to faculty to administration.

Similar to prior data,^2^, ^3^, ^4^ we found that required POCUS education was more commonly implemented into the preclinical years, with more elective or optional coursework being offered during the 3rd and 4th years of medical school. This makes sense as to understand POCUS, the operator needs to have a strong understanding of anatomy. Additionally, evidence‐based research shows that POCUS integration into UME improves anatomy education and improves the physical examination of abdominal, reproductive, cardiovascular, and renal structures.^6^, ^7^ The preclinical years may be easier from an implementation standpoint, where students tend to be together for learning compared to the clinical years of medical school, where students may spread across a state, limiting the ability to teach POCUS broadly. Medical schools may also lack POCUS‐trained instructors or the infrastructure needed to require POCUS beyond the preclinical years.

Interestingly, we found that the number of campuses a school has, which could be seen as a barrier as multiple campuses require more coordination and resources,^14^ did not impact the likelihood of having a POCUS curriculum. Instead, the rates of having a POCUS curriculum were the same 65% for multisite institutions versus 66% for single campus institutions. Prior barriers identified for implementing POCUS in UME included lack of trained faculty and lack of resources including equipment.^2^, ^3^, ^4^ In this study, we found that many schools were using a broad range of faculty to teach students. We also found that a large percent of schools (74%, 59/80) were using handheld POCUS machines, which provide a more cost‐effective alternative to cart‐based machines and are more portable. This was an increase from prior data finding that 46 schools used handheld machines.^4^ This may be a potential reason we saw schools with multiple campuses and overall, more schools were able to successfully implement a curriculum as handheld machines can significantly decrease the cost of equipment without sacrificing diagnostic accuracy.^15^

We found that only 49 (61%) schools were assessing students' progress or understanding of POCUS. Compared to data collected 5 years ago, this number did not change significantly, where 48 schools were assessing students' knowledge and skill at that time. While exposing students to POCUS is an important first step for their comfort and knowledge with POCUS, implementing timely assessments is necessary to evaluate competency‐based medical education to ensure that students are learning and progressing.^16^ Lack of assessments, which is seen in this study and in prior studies, may be due in part to a lack of national standards on what should be taught and how POCUS competence should be assessed in UME.^17^ Additionally, implementing assessments can be time‐ and resource‐intensive. Moving forward beyond schools implementing POCUS, there is a need to develop and implement competency‐based assessments designed for POCUS in UME to ensure students are learning and able to take this knowledge and skill into residency training where they can continue to build on this foundation.

### Limitations

This study had several limitations that may affect its generalizability. The data collected in this study was dependent on survey responses. Although we had a high response rate at 84%, we were still missing data from 25 schools. We did not assess osteopathic schools, which should be considered for future studies as a large portion of medical students matriculate from osteopathic schools. Additionally, despite making multiple attempts to contact the person most knowledgeable of the POCUS curriculum at each school using multiple methods, it is possible that representatives from a school less involved with all the details of the curriculum filled out the survey. The data presented in this survey is not representative of all US allopathic medical schools, and some relevant details could have been missed in the survey responses. Regardless, the data presented in this study is in line with prior literature^2^, ^3^, ^4^ and the addition of the missing data likely would not have altered the results.

## Conclusion

In conclusion, the implementation of POCUS in UME continues to expand, with 66% of schools having an approved curriculum, with most schools requiring POCUS in the preclinical phases of medical school. Progress has been seen over the last 5 years, with more schools implementing POCUS in UME and more offering a 4‐year longitudinal curriculum. However, there remains a need for evidence‐based national guidelines on how to best implement POCUS in UME and evaluate competency.

## Supporting information


**Supplemental Appendix 1** Point‐of‐care ultrasound survey distributed in 2024 to faculty from allopathic medical schools.

## Data Availability

The data that support the findings of this study are available from the corresponding author upon reasonable request.
