# Supplementary material for: Point‐of‐Care Ultrasound in Undergraduate Medical Education: A National Survey
Source: J Ultrasound Med. 2025 Aug 4;44(12):2335–40. doi: 10.1002/jum.70021 (PMC12605648; doi:10.1002/jum.70021)
Supplement: Supplementary file 1 — Supplemental Appendix 1 Point‐of‐care ultrasound survey distributed in 2024 to faculty from allopathic medical schools. [file JUM-44-2335-s001.pdf]

Demographics

What is your name?
Collecting this in case we have multiple responses from a single medical school.

What is your academic position? (choose the position that fits you best)

- ◯ Dean of Education or Curriculum Design
- ◯ Point of Care Ultrasound Faculty (ie Clinical Ultrasound Director for UME or GME, POCUS Division Director or POCUS Faculty)
- ◯ Other (please list your title if it does not fit the top 2 categories)

What is the name of your institution?

How many campuses does your school have?

Ultrasound in Undergraduate Medical Education

The below questions are pertaining to POCUS in Undergraduate Medical Education (medical student).

Does your medical school have a POCUS curriculum that has been **approved** by your school's curriculum committee? (including elective or required elements)

- ◯ Yes
- ◯ No

Is the POCUS curriculum:

- ◯ Required
- ◯ Optional or elective
- ◯ Both

Ultrasound Curriculum

What POCUS topics are you teaching medical students?
Select all that apply:

- ☐ Fundamentals of US (i.e. physics, knobology, scanning for physical exam skills teaching, etc.)
- ☐ Diagnostic US
- ☐ Procedural US

What aspects of **diagnostic US** are you teaching?
Select all that apply.

- ☐ Aorta
- ☐ eFAST
- ☐ Cardiac and IVC
- ☐ Gallbladder/RUQ
- ☐ Kidneys and bladder
- ☐ Lung
- ☐ Musculoskeletal
- ☐ Obstetric
- ☐ Ocular

- [ ] Soft tissue
- [ ] RUSH: US in Hypotension
- [ ] US in undifferentiated dyspnea
- [ ] Other
- [ ] All the above

What **procedural US** skills are you teaching?

- [ ] Nerve blocks
- [ ] Paracentesis
- [ ] Thoracentesis
- [ ] Vascular access
- [ ] Other
- [ ] All the above

When in the UME curriculum are you implementing POCUS?

	Select all that apply<br>Required	Select all that apply<br>Elective	Select all that apply<br>Neither
First year	☐	☐	☐
Second year	☐	☐	☐
Third year	☐	☐	☐
Fourth year	☐	☐	☐

Where are you implementing POCUS in the curriculum?
Select all that apply.

	Required	Optional/Elective	Neither
Anatomy	☐	☐	☐
Physical exam	☐	☐	☐
Physiology	☐	☐	☐
Pathology	☐	☐	☐
Clerkships	☐	☐	☐
Sub-internship	☐	☐	☐
Dedicated US Course	☐	☐	☐

What medical specialties are involved in teaching POCUS in UME? Select all that apply.

- ☐ Anesthesia
- ☐ Cardiology
- ☐ Critical Care
- ☐ Emergency medicine
- ☐ Family Medicine
- ☐ Internal medicine
- ☐ OB/GYN
- ☐ Pediatrics
- ☐ Radiology
- ☐ Surgery
- ☐ Anatomy PhD
- ☐ US Technologists
- ☐ Other

What equipment are you using to teach POCUS?

- ◯ Handheld ultrasounds
- ◯ Cart-based ultrasounds
- ◯ Both

Assessment of Learners

Does your curriculum assess its learner's progress or understanding of POCUS?

- ◯ Yes
- ◯ No

How are you assessing student's **knowledge**?
Select all that apply.

- ☐ Reviewing submitted US images
- ☐ Test/quiz questions
- ☐ Hands-on assessment (OSCE/SDOT)
- ☐ We are not assessing knowledge

How are you assessing student's **ability to perform US**?

- ☐ Hands-on assessment (OSCE/SDOT)
- ☐ Simulation
- ☐ Reviewing submitted US images
- ☐ We are not assessing ability to perform US

POCUS in GME

The following 2 questions pertain to your schools GME (residency and fellowship) curricula and NOT UME.

What specialities in **graduate medical education** (residency/fellowship) at your school have a required POCUS curriculum?

- ☐ Emergency Medicine
- ☐ Cardiology
- ☐ Surgery
- ☐ Pediatrics (excluding pediatric emergency medicine)
- ☐ Critical Care
- ☐ Internal Medicine (excluding subspecialties)
- ☐ OB/GYN
- ☐ Ophthalmology
- ☐ Orthopedics
- ☐ Neurology
- ☐ Anesthesiology
- ☐ Family Medicine
- ☐ Sports Medicine
- ☐ I do not know
- ☐ None that I am aware of

Does your school have a **required** core POCUS curriculum for GME? (where multiple specialties participate in the same core POCUS curriculum)

- ○ Yes
- ○ No
- ○ I do not know

Powered by Qualtrics
